# Supplementary material for: Coupling Molecular and Cellular Dynamics in a Large-Scale Monte Carlo Simulation
Source: Int J Mol Sci. 2025 Nov 5;26(21):10763. doi: 10.3390/ijms262110763 (PMC12609721; doi:10.3390/ijms262110763)
Supplement: Supplementary file 1 [file ijms-26-10763-s001.zip › Figure_S1.pdf]

A

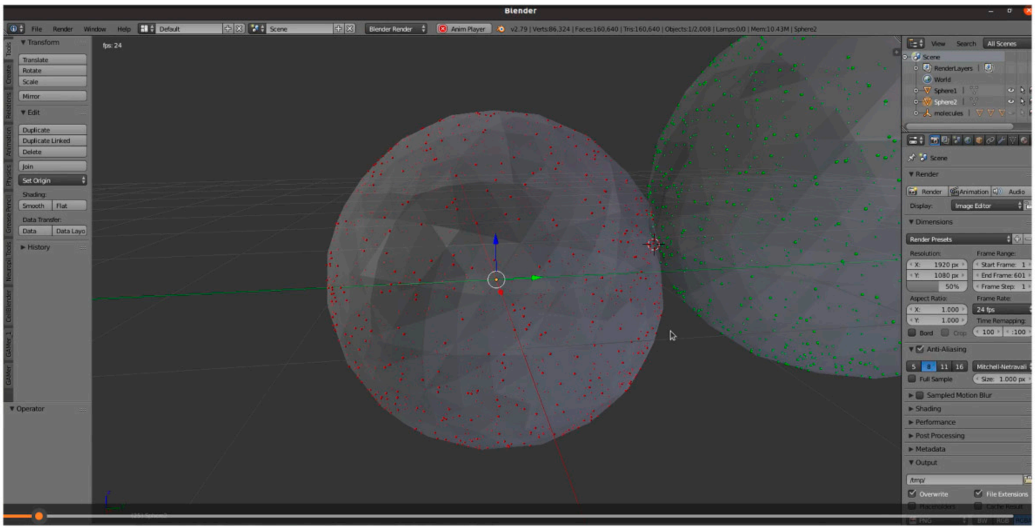

B

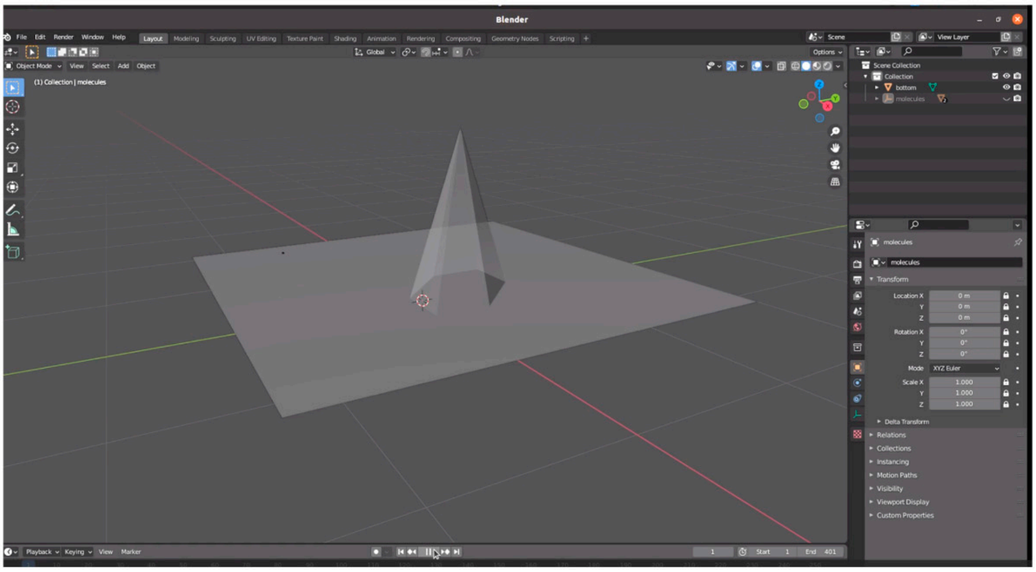

Figure S1. The user interface of the simulation. The simulation uses Blender and CellBlender interfaces for construction of geometry and visualization. Running the simulation is preferentially conducted from the command line. (A) MCell3 interface. (B) MCell4 interface.
