# Supplementary material for: Coupling Molecular and Cellular Dynamics in a Large-Scale Monte Carlo Simulation
Source: Int J Mol Sci. 2025 Nov 5;26(21):10763. doi: 10.3390/ijms262110763 (PMC12609721; doi:10.3390/ijms262110763)
Supplement: Supplementary file 1 [file ijms-26-10763-s001.zip › Supplementary Table S1-ijms-3885317.pdf]

Supplementary Table S1. Physical parameters of cell related simulations.

Parameter*	Cell-cell simulation (Fig. 2E)		Cell spreading simulation (Fig. 3C)		Units
	**Right cell1**	**Left cell**	**Cell**	**Surface**	
Membrane stretching modulus	10	Inf	10	Inf	μm^{-2}
Internal Pressure	0.01	0	** actin polymerization at 10 nm*s^{-1}	0	μm^{-3}
Molecular diffusion	NA	NA	10^{-5}	0	μm^{2}*s^{-1}
Receptor-ligand interaction Radius	NA	NA	0.005 – 0.05		µm
Number of molecules	NA	NA	250,000	250,000	Molecules
Grid size	642	162	12,459	4,900	Vertices
Physical mesh size	4π	4π	~$2\pi 6^2$	15x15	μm^2

* Physical parameters for both simulations: Energy is in units of 1k_BT, iteration time = 1ms, no bending applied; ** Internal pressure in the cell spreading simulation is due to (implicit) actin polymerization, applied at the edges of the cell protrusions at a velocity of 10 nm*s^{-1}.
