# Supplementary figures and images for: Coupling Molecular and Cellular Dynamics in a Large-Scale Monte Carlo Simulation
Source: Int J Mol Sci. 2025 Nov 5;26(21):10763. doi: 10.3390/ijms262110763 (PMC12609721; doi:10.3390/ijms262110763)

Movie M1

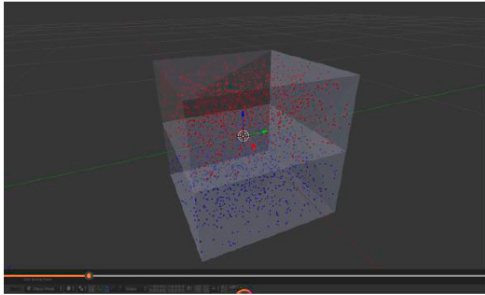

Movie M2

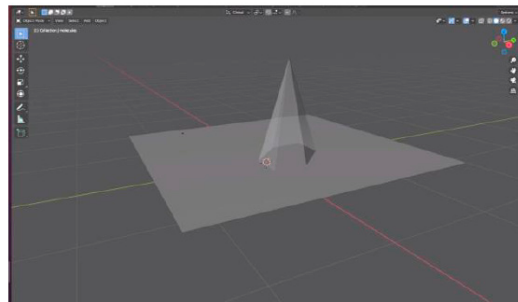

Movie M3

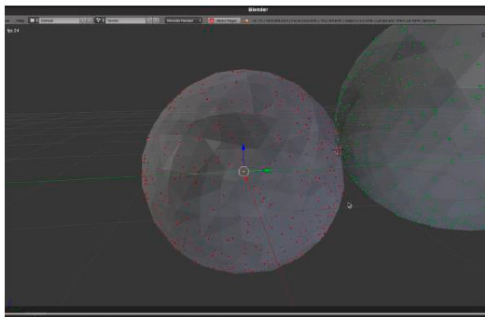

Movie M4

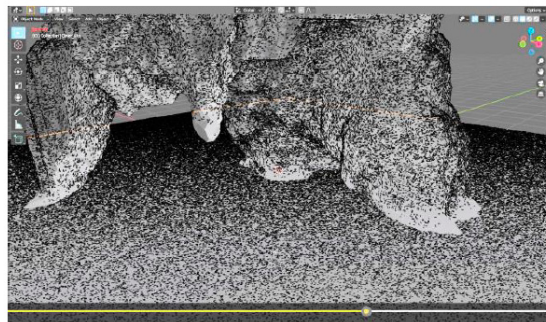

Screenshots from the 4 supplementary movies

Supplement: Supplementary file 1 [file ijms-26-10763-s001.zip › Screenshots from the 4 supplementary movies.pdf]
